# Groundwater quality assessment for drinking and agriculture purposes in Abhar city, Iran

**DOI:** 10.1016/j.dib.2018.05.096

**Published:** 2018-05-23

**Authors:** Khadijeh Jafari, Farzaneh Baghal Asghari, Edris Hoseinzadeh, Zahra Heidari, Majid Radfard, Hossein Najafi Saleh, Hossein Faraji

**Affiliations:** a Department of Environmental Health Engineering, Faculty of Health, Hormozgan University of Medical Sciences, Bandar Abbas, Iran; b Department of Environmental Health, School of public Health, Tehran University of Medical Sciences, Tehran, Iran; c Department of Environmental Health, Faculty of Medical Sciences, Tarbiat Modares University, Tehran, Iran; d Bachelor of Environmental Health, Department of Health, Isfahan University of Medical Sciences, Isfahan, Iran; e Health Research Center, Baqiyatallah University of Medical Sciences, Tehran, Iran; f Department of Environmental Health Engineering, Torbat Heydariyeh University of Medical Sciences, Torbat Heydariyeh, Iran; g Students Research Committee, Hamadan University of Medical Sciences, Hamadan, Iran

**Keywords:** Groundwater, Agricultural indicator, Drinking indicator, Abhar, Iran

## Abstract

The main objective of this study is to assess the quality of groundwater for drinking consume and agriculture purposes in abhar city. The analytical results shows higher concentration of electrical conductivity (100%), total hardness (66.7%), total dissolved solids (40%), magnesium (23%), Sulfate (13.3%) which indicates signs of deterioration as per WHO and Iranian standards for drinking consume. Agricultural index, in terms of the hardness index, 73.3% of the samples in hard water category and 73.3% in sodium content were classified as good. Therefore, the main problem in the agricultural sector was the total hardness Water was estimated. For the RSC index, all 100% of the samples were desirable. In the physicochemical parameters of drinking water, 100% of the samples were undesirable in terms of electrical conductivity and 100% of the samples were desirable for sodium and chlorine parameters. Therefore, the main water problem in Abhar is related to electrical conductivity and water total hardness.

**Specifications Table**

**Table:** 

Subject area	Chemistry
More specific subject area	Describe narrower subject area
Type of data	Tables and figure
How data was acquired	EC, pH and chloride were analyzed using multiple parameters ion meter model Thermo Orion 5 Star. Sulfate (SO_4_^−2^) was measured using a double beam UV–Vis spectrophotometer model Perkin Elmer Lambda 35 by turbid-metric, stannous chloride, and molybdo silicate, respectively. Sodium, calcium and magnesium were analyzed using flame photometer model CL-378 (Elico, India). Total hardness was determined by EDTA titrimetric method. TDS was measured gravimetrically). Agricultural indicator such as SAR, RSC, PI, KR, MH, and PS, % Na, SSP and TH were calculated using the Their formulas.
Data format	Raw, Analyzed
Experimental factors	All water samples in polyethylene bottles were stored in a dark place at room temperature until the metals analysis
Experimental features	Determine the content levels of physical and chemical parameters
Data source location	Abhar, Zanjan province,Iran
Data accessibility	Data are included in this article

**Value of data**

• Determination of the Agricultural and drinking water indices including SAR, %Na, SSP,MH, KR,RSC, EC, Ca^2+^, Mg^+2^,pH, TDS, TH, HCO_3_^-^, Na^+^, K^+^, Cl^−^, and SO_4_^2−^ in ground water was conducted in Abhar city, Iran.
• The level of EC, TDS and total hardness in the water samples indicates that maximum of them are unsuitable for drinking consume.
• Agricultural indices such as SAR and SSP indicated 100, 90% of samples in the studied area had SAR and SSP values within the excellent category respectively for irrigation purposes.
• Data of this study can help to better understand the quality of groundwater in this area.
• The present data d study recommends that regular monitoring of groundwater is essential to avoid major environmental threat

## 1 Data

Summary of the physical and chemical variables for the collected groundwater samples were presented in Table 1, The analytical results shows higher concentration of electrical conductivity (100%), total hardness (66.7%), total dissolved solids (40%), magnesium (23%), Soulphat (13.3%) which indicates signs of deterioration as per WHO and Iranian standards for drinking consume Table 2.

**Table 1:** Water level and physico-chemical analyses of groundwater samples of study area collected during 2016 year.

**Well no**	**pH**	**Na**^**+**^	**Mg**^**2+**^	**Ca**^**2+**^	**Cl**^**−**^	**K**^**+**^	**CO**_**3**_^**−**^	**HCO**_**3**_^**−**^	**SO**_**4**_^**−**^	**TDS**	**EC**	**T.H**
		**(mg/L)**	**(mg/L)**	**(mg/L)**	**(mg/L)**	**(mg/L)**	**(mg/L)**	**(mg/L)**	**(mg/L)**	**(mg/l)**	**(μmhos/cm)**	**(mg/l)**
P1	7.26	77.51	32.55	81.4	63.55	1.95	0	419.68	68.16	640	1045	338
P2	7.54	36.11	31.10	42.4	19.53	1.56	0	273.28	57.12	400	645	234
P3	7.66	119.37	19.00	58.2	60.71	1.17	0	195.2	220.8	640	998	224
P4	5.98	117.07	29.65	145.4	37.28	2.73	0	580.72	190.08	950	1516	486
P5	7.02	45.08	23.35	49.6	23.43	1.17	0	248.88	74.88	410	658	220
P6	6.97	73.37	33.03	50.4	35.15	2.34	0	346.48	83.04	540	875	262
P7	7.16	121.9	29.65	67.8	47.22	1.17	0	195.2	310.08	740	1153	292
P8	7.34	54.05	16.58	56.6	24.50	1.17	0	297.68	45.12	420	676	210
P9	7.27	55.2	25.29	68.6	45.09	1.95	0	307.44	74.88	510	817	276
P10	7.21	59.8	32.55	57.4	31.24	3.12	0	341.6	80.16	530	848	278
P11	7.58	64.63	30.61	44.8	27.34	2.73	0	307.44	81.12	490	786	238
P12	7.13	80.96	27.71	98.2	66.74	1.95	0	458.72	60	680	1098	360
P13	7.36	23.23	34.00	42.4	15.62	1.17	0	263.52	52.8	370	613	246
P14	7.22	62.1	26.26	55.2	32.31	1.56	0	326.96	61.92	490	788	246
P15	7.31	37.95	20.45	68.6	29.47	1.56	0	307.44	39.84	430	698	256
P16	7.26	67.39	21.90	63	27.34	1.56	0	297.68	104.16	510	815	248
P17	7.35	82.57	14.64	40.8	29.47	1.56	0	263.52	79.2	440	706	162
P18	7.32	25.99	9.68	39.2	13.85	0.78	0	180.56	24.96	250	402	138
P19	7.43	32.2	7.74	48	22.72	0.78	0	195.2	26.88	280	458	152
P20	7.51	41.4	7.74	46.4	14.56	1.17	0	195.2	52.8	310	494	148
P21	7.47	114.77	24.32	81.4	47.22	1.56	0	258.64	260.16	730	1143	304
P22	7.44	28.29	8.71	41.6	15.62	0.78	0	185.44	24	260	417	140
P23	7.33	105.8	20.45	59	34.44	1.17	0	278.16	172.8	610	962	232
P24	7.46	21.85	10.65	47.2	10.65	0.78	0	204.96	23.04	270	434	162
P25	7.07	46.92	21.42	60.6	31.24	1.56	0	287.92	57.12	440	707	240
P26	7.32	33.81	7.74	52	16.69	0.78	0	165.92	70.08	310	490	162
P27	7.24	29.9	10.16	57.4	25.56	0.78	0	190.32	54.24	320	518	186
P28	7.37	78.66	14.16	41.6	31.24	0.78	0	200.08	116.16	440	693	162
P29	7.36	126.73	33.03	65.4	51.12	1.95	0	263.52	271.2	760	1190	300
P30	7.42	25.3	8.23	39.2	15.62	0.78	0	175.68	19.2	240	390	132
**Min**	6.0	21.9	7.7	39.2	10.7	0.8	0	165.9	19.2	240.0	390.0	132.0
**Max**	7.7	126.7	34.0	145.4	66.7	3.1	0	580.7	310.1	950.0	1516.0	486.0
**Ave**	7.3	63.0	21.1	59.0	31.5	1.5	0	273.8	95.2	480.3	767.8	234.5
**SD**	0.29	33.30	9.25	21.54	15.16	0.65	0	92.46	78.88	176.78	277.06	77.64

**Table 2:** Quality of groundwater samples from Abhar city for drinking purpose compared with WHO and Iranian standard (1053) [1], [2], [3], [4], [5], [6], [7].

**Parameter**	**Desirable limit**	**2016 Year samples(%)**	
		**Within limits**	**Exceed limits**
**pH**	6.5–8.5	96.7	3.3
**EC**	300 (μmhos/cm)	0	100
**TDS**	500 (mg/L)	60	40
**Total hardness**	200 (mg/L)	33.3	66.7
**SO**_**4**_^**2−**^	200 (mg/L)	86.7	13.3
**Cl**^**−**^	250 (mg/L)	100	0
**Ca**^**2+**^	75 (mg/L)	86.7	13.3
**Mg**^**2+**^	30 (mg/L)	76.7	23.3
**Na**^**+**^	200 (mg/L)	100	0

As shown in Table 3, Table 4, the calculated SAR, SSP, PI, MH, KR and Na% values were compared with the groundwater quality classification, where 100,90% of samples in the studied area had SAR and SSP values within the excellent category respectively for irrigation purposes.

**Table 3:** Calculation of RSC, PI, KR, MH, Na%, SAR and SSP of groundwater for 2016 year.

Well ID	RSC	PI	KR	MH	Na%	SAR	SSP
P1	0.12	59.16	0.50	39.79	33.60	1.83	33.27
P2	−0.21	58.89	0.33	54.80	25.56	1.03	25.08
P3	−1.28	72.17	1.16	35.04	53.81	3.47	53.67
P4	−0.2	55.20	0.52	25.21	34.68	2.31	34.37
P5	−0.33	62.48	0.44	43.76	31.09	1.32	30.77
P6	0.43	66.03	0.61	52.00	38.24	1.97	37.80
P7	−2.64	63.63	0.91	41.95	47.72	3.10	47.58
P8	0.68	69.60	0.56	32.62	36.17	1.62	35.88
P9	−0.48	58.65	0.43	37.86	30.74	1.44	30.30
P10	0.04	60.86	0.47	48.38	32.52	1.56	31.86
P11	0.27	66.69	0.59	53.04	37.65	1.82	37.07
P12	0.32	58.42	0.49	31.81	33.15	1.86	32.84
P13	−0.61	51.99	0.20	57.00	17.42	0.64	17.00
P14	0.43	65.73	0.55	44.02	35.72	1.72	35.39
P15	−0.08	57.53	0.32	33.01	24.82	1.03	24.37
P16	−0.08	65.13	0.59	36.49	37.45	1.86	37.14
P17	1.07	82.87	1.10	37.23	52.76	2.82	52.49
P18	0.2	73.28	0.41	28.99	29.41	0.96	29.05
P19	0.16	71.82	0.46	21.05	31.84	1.14	31.53
P20	0.24	75.40	0.61	21.62	38.20	1.48	37.82
P21	−1.84	63.68	0.82	33.06	45.27	2.86	45.08
P22	0.24	73.79	0.44	25.71	30.86	1.04	30.52
P23	−0.08	72.89	0.99	36.42	49.95	3.02	49.78
P24	0.12	66.42	0.29	27.16	23.04	0.75	22.67
P25	−0.08	61.59	0.43	36.88	30.23	1.32	29.82
P26	−0.52	66.23	0.45	19.75	31.50	1.15	31.21
P27	−0.59	61.20	0.35	22.64	26.24	0.95	25.95
P28	0.03	78.43	1.05	36.00	51.42	2.68	51.27
P29	−1.68	65.93	0.92	45.50	48.10	3.18	47.87
P30	0.24	74.79	0.42	25.76	29.79	0.96	29.41
**Min**	−2.64	51.99	0.20	19.75	17.42	0.64	17.00
**Max**	1.07	82.87	1.16	57.00	53.81	3.47	53.67
**Ave**	−0.20	66.02	0.58	36.15	35.63	1.76	35.30
**SD**	0.78	7.28	0.26	10.40	9.31	0.81	9.38

**Table 4:** Classification of groundwater sample for irrigation use on the basic of EC, SAR, RSC, KR, SSP, PI, MH, Na%, T.H.

**Parameters**	**Range**	**Water class**	**Samples (%)**
			**2016 Year**
**EC**	<250	Excellent	Nil
	250–750	Good	53.3
	750–2250	Permissible	46.7
	>2250	Doubtful	Nil
**SAR**	0–10	Excellent	100
	10–18	Good	Nil
	18–26	Doubtful	Nil
	>26	Unsuitable	Nil
**RSC**	<1.25	Good	100
	1.25–2.5	Doubtful	Nil
	>2.5	Unsuitable	Nil
**KR**	<1	suitable	90
	1–2	Marginal suitable	10
	>2	Unsuitable	Nil
**SSP**	<50	Good	90
	>50	Unsuitable	10
**PI**	>75	Class-I	10
	25–75	Class-II	90
	<25	Class-III	Nil
**MH**	<50	Suitable	86.7
	>50	Harmful &Unsuitable	13.3
**Na%**	<20	Excellent	3.3
	20–40	Good	73.3
	40–60	Permissible	23.4
	60–80	Doubtful	Nil
	>80	Unsuitable	Nil
**T.H**	<75	Soft	Nil
	75–150	Moderately Hard	13.3
	150–300	Hard	73.3
	>300	Very Hard	13.4

## 2 Experimental design, materials and methods

### 2.1 Study area description

**Fig. 1:**
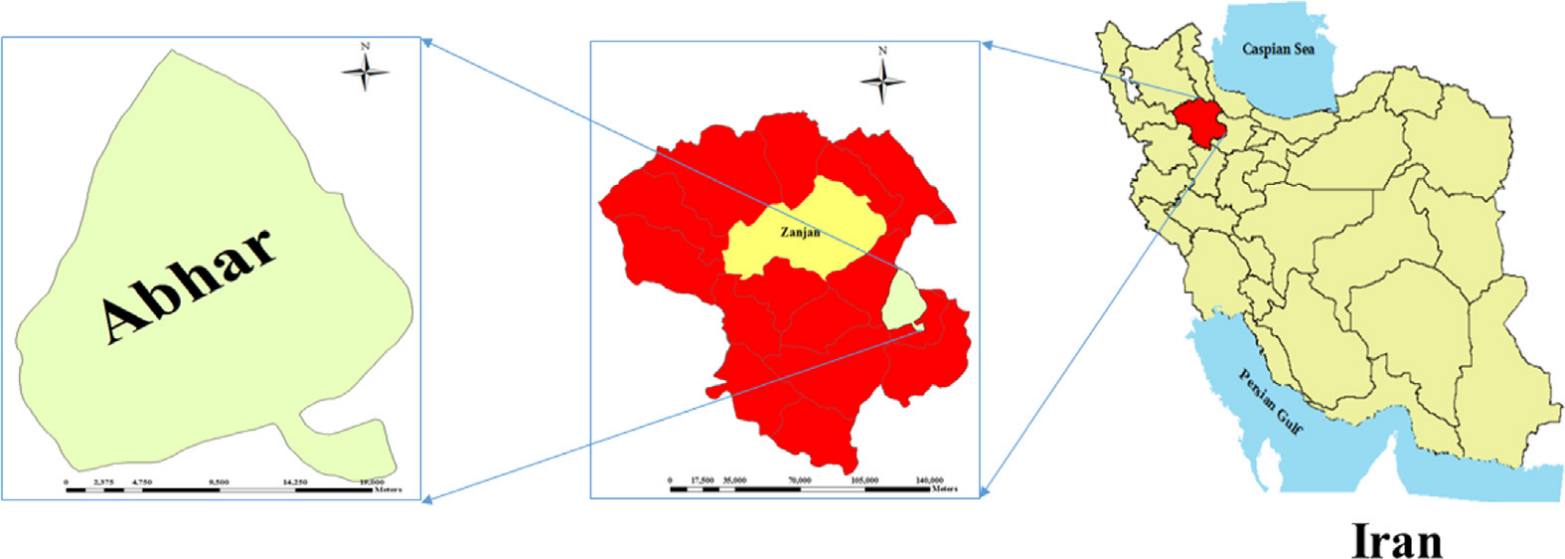
Location of the study area in Abhar city, Zanjan province, Iran.

Abhar is one of the cities of Zanjan province and Abhar city center. The city with 99,285 people in 2016 is considered as the second most populated city of Zanjan province after Zanjan city. The height of Abhar city is 1540 m. The maximum relative humidity in the city is 94.4% and at least 23.3%. The average annual rainfall is 300 mm. This area is considered as a semi-cold and dry in Iran country [8] (Fig. 1).

### 2.2 Determination of the physico-chemical parameters concentration and agricultural indicators

In order to assess the physico-chemical parameters, a total of 30 samples taken from Abhar County (Fig. 1). Water samples were collected in a plastic container of 1-L capacity for detailed chemical analysis from all observation wells. These containers were washed thoroughly with distilled water and dried before being filled with water samples. The containers were numbered serially along with a proper record of well/sample location, date, static water level, and prior to the sampling. Groundwater samples were collected after the well was subjected to pumping for at least 5–10 min to obtain the composite sample. The pH and EC of the groundwater of the wells were measured by using HACH HQ40d and its in situ values are recorded [9], [10], [11], [13], [14], [15]. The samples were collected and stored below 4 °C and analyzed in the Centre for Water Resources Development and Management (CWRDM). Water samples collected in the field for chemical constituents, such as TDS, TH, Ca^2+^, Mg^2+^, CO_3_^2−^, HCO_3_^−^, Na^+^, Cl^−^ and SO_4_^2−^, were analyzed following the BIS standard. EC, pH and chloride (Cl^-^) were analyzed using multiple parameters ion meter model Thermo Orion 5 Star. Sulfate (SO4 _4_^−2^) was measured using a double beam UV–Vis spectrophotometer model Perkin Elmer Lambda 35 by turbid-metric, stannous chloride, and molybdosilicate, respectively. Sodium (Na^+^), calcium (Ca^2+^) and magnesium (Mg^2+^) were analyzed using flame photometer model CL-378 (Elico, India). Total hardness was determined by EDTA titrimetric method [1], [16], [17], [18]. TDS was measured gravimetrically and Agricultural indicator such as SAR, RSC, PI, KR, and MH, % Na, SSP and TH were calculated by their formulas presented in Table 5.

**Table 5:** Summary of water quality indices in present study [1].

**Indices**	**Formula**
Residual sodium carbonate (RSC)	RSC=(CO_3_^2−^ + HCO_3_^−^)+(Ca^2+^ + Mg^2+^)
Permeability index (PI)	$\mathrm{PI}=\frac{\mathrm{Na}+K+\sqrt{\mathrm{HCO}3}}{\mathrm{Ca}+\mathrm{Mg}+\mathrm{Na}+K}\times 100$
Kelly׳s ratio (KR)	$\mathrm{KR}=\frac{\mathrm{Na}}{\mathrm{Ca}+\mathrm{Mg}}$
Magnesium hazard(MH)	$\mathrm{MH}=\frac{\mathrm{Mg}}{\mathrm{Ca}+\mathrm{Mg}}\times 100\;$
Sodium percentage (Na %)	$\mathrm{Na}\%=\frac{\mathrm{Na}+K}{\mathrm{Ca}+\mathrm{Mg}+\mathrm{Na}+K}\times 100$
Sodium adsorption ratio (SAR)	$\mathrm{SAR}=\frac{\mathrm{Na}}{\sqrt{(\mathrm{Ca}+\mathrm{Mg})/2}}\times 100$
Soluble sodium percentage (SSP)	$\mathrm{SSP}=\frac{\mathrm{Na}}{\mathrm{Ca}+\mathrm{Mg}+\mathrm{Na}}\times 100\;$
